# Insights about multi-targeting and synergistic neuromodulators in Ayurvedic herbs against epilepsy: integrated computational studies on drug-target and protein-protein interaction networks

**DOI:** 10.1038/s41598-019-46715-6

**Published:** 2019-07-22

**Authors:** Neha Choudhary, Vikram Singh

**Affiliations:** Centre for Computational Biology and Bioinformatics, School of Life Sciences, Central University of Himachal Pradesh, Dharamshala, 176206 India

**Keywords:** Statistical methods, Data integration, Systems analysis, Predictive medicine, Epilepsy

## Abstract

Epilepsy, that comprises a wide spectrum of neuronal disorders and accounts for about one percent of global disease burden affecting people of all age groups, is recognised as *apasmara* in the traditional medicinal system of Indian antiquity commonly known as Ayurveda. Towards exploring the molecular level complex regulatory mechanisms of 63 anti-epileptic Ayurvedic herbs and thoroughly examining the multi-targeting and synergistic potential of 349 drug-like phytochemicals (DPCs) found therein, in this study, we develop an integrated computational framework comprising of network pharmacology and molecular docking studies. Neuromodulatory prospects of anti-epileptic herbs are probed and, as a special case study, DPCs that can regulate metabotropic glutamate receptors (mGluRs) are inspected. A novel methodology to screen and systematically analyse the DPCs having similar neuromodulatory potential *vis-à-vis* DrugBank compounds (NeuMoDs) is developed and 11 NeuMoDs are reported. A repertoire of 74 DPCs having poly-pharmacological similarity with anti-epileptic DrugBank compounds and those under clinical trials is also reported. Further, high-confidence PPI-network specific to epileptic protein-targets is developed and the potential of DPCs to regulate its functional modules is investigated. We believe that the presented schema can open-up exhaustive explorations of indigenous herbs towards meticulous identification of clinically relevant DPCs against various diseases and disorders.

## Introduction

Epilepsy (EP) is one of the oldest known conditions of neuronal disorders across the globe, with a history dating back to 4,000 BC of misconception, unawareness and humiliation^1^. This neurological disorder has been one of the most researched medical condition mainly due to the complex morbidity associations with substantially high mortality rates^2,3^. High mortality is considered to be the consequence of underlying disorders associated with the disease that may include non-epilepsy related other factors also^4,5^. EP is amongst the top three leading contributors to global burden of neurological disorders, affecting about 65 million people worldwide with widely varying prevalence and incidence throughout the world^6^. In low and middle-income countries where 80–90% of epilepsy patients receive no treatment at all, epilepsy-related mortality is 2.6 fold higher than the general population^7,8^. The prevalence of this disorder in South East Asia is 2–10 persons per 1,000 population of which more than 50% of disability-adjusted life years (DALYs) are contributed from India. Further, the effect of this disorder on children is quite disturbing. Compared to the general population, 10 times higher death risk is reported in case of children^9^. For the childhood epilepsy, complex interactions among underlying epileptic co-morbidities emerging in the developing young brain constitute the major treatment challenge^10^.

EP is a chronic neuronal disorder of the brain that is characterized by recurrent and spontaneous seizures (epileptic seizures) leading to events of involuntary movement of the body or body parts. Clinical manifestations of an epileptic seizure are accredited to the synchronous neuronal activity within the brain^11^. Based on the aetiology, EP can be categorized into two categories: symptomatic and idiopathic EP. While symptomatic EP could be remote (conditions attributed to head injury, stroke, CNS infection) or progressive (brain tumor and degenerative disease) in nature, idiopathic has no identifiable cause. It should be taken into consideration that the complex behavior of EP is also attributed to the psychological, social and economic backgrounds^6^. Although remarkable advancements towards the understanding and treatment of EP have been achieved in past, existence of neurological and psychiatric co-morbidities undermine the current treatment strategies of EP^12^. Further, the conventional approach of “one-drug – one-target – one-disease” treatment schema seems to be unsuccessful in providing satisfactory results. Since the locus of the seizure in majority of EP patients is not confined to a single region of the cortex, even surgery is not opted as a treatment strategy in such cases. Also, the use of new generation antiepileptic drugs is less successful in completely controlling the seizures, thus providing scope for improvements in current drug-development procedures^13^. Drug-resistant seizures have also been observed in nearly one-third of epileptic patients^14^. Considering all these factors into account, network medicine approach for the treatment of EP could be of significant importance. Owing to their suitability in the management of complex diseases including drug-resistant cases, ideas that can integrate the concepts of network biology with polypharmacology are drawing attention of the scientific community^15^.

Identification of drug-protein interactions is considered as a fundamental step towards the drug discovery approaches as it deciphers the pharmacology of a drug molecule and aids information about its therapeutic efficacy. In the era of big data revolution that has also resulted in an explosion of biological data, information of known drug-target pairs has allowed the research community to explore computational methods for predicting the unknown compound-protein interactions. Variety of computational methods^16,17^ and databases^18–21^ for the *in-silico* prediction of drug targets are available and new initiatives towards better predictions are being continuously undertaken. Concepts of network properties have also been applied to predict, analyze and suggest potential new drug-target interactions^17,18^. Since the activities and functions of protein molecules are often regulated by the other proteins present in the system with which it is interacting, consideration of PPI (protein-protein interaction) networks in the analysis provides substantial enhancements towards deciphering the comprehensive systems-level effects of the compound under investigation. Owing to the great expense of experimental approaches in terms of both time consumption and money, efficient and effective *in-silico* methods for the prediction of PPIs have been developed^22–24^

Since herbal medicines that are one of the most popular forms of complementary and alternative medicines (CAMs) are effective and safe in nature, their usage in the treatment of neurological diseases and disorders is also gaining importance. Herbal formulations exert their poly-pharmacological effects by acting on the multiple targets using its multi-component framework^25^. Trend of the treatment of EP using CAMs is not new and nearly 50% of the patients are still using this strategy for the treatment^26,27^. Ayurveda, the ancient Indian medicinal system defines EP as *Apasmara*: where *apa* refers to “negation” and *smara* as “consciousness”^28^. Ayurveda describes the use of various plants and plant-based formulations for the management of neurological conditions like migraine, epilepsy, Alzheimer’s, anxiety, Parkinson’s, depression etc.^29–31^. Due to rapid advancements in the field of chemical and synthetic biology, the network pharmacological paradigm of drug discovery is showing potential to be efficient and effective in exploring the molecular mechanisms of various herbs like *Piper longum*^32^, herbal formulae’s like Liu-Wei-Di-Huang pill^33^ and therapeutic molecules like cannabidiol^34^ etc. In the direction of providing better therapeutic effects, concepts of network pharmacology are being utilized to explore potential combinations of drugs^35^. It is a poly-pharmacological approach which relies on the principle of “multiple compounds – multiple-targets” approach for the disease treatment and represents the holistic mechanism of the drugs, targets and disease in a systematic manner.

**Figure 1:**
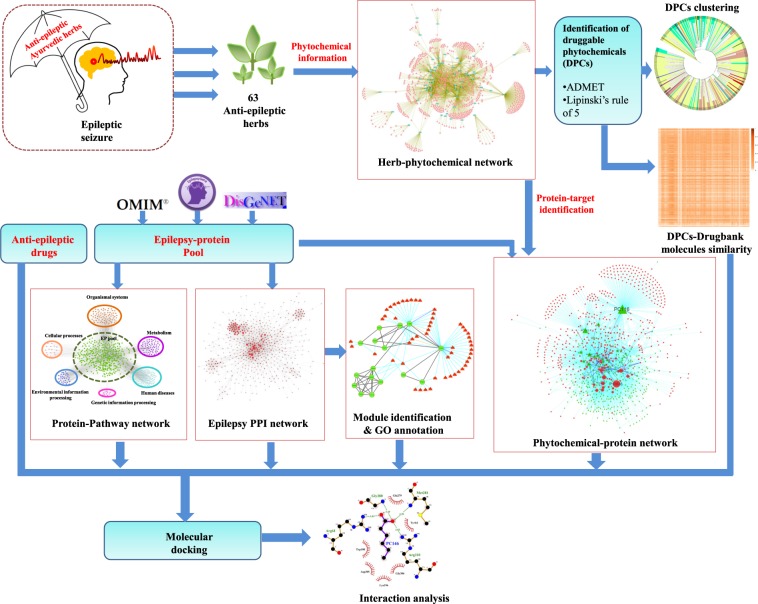
Detailed workflow of the present study.

Therefore, in the present study, we have first identified the extensively used anti-epileptic herbs of Ayurveda and then their poly-pharmacological evaluation was carried out using the approach of network pharmacology. For this, we have manually reviewed and identified the anti-epileptic herbs of Ayurveda from various literature resources. A comprehensive dataset of phytochemicals present in identified herbs was prepared using the information present in public databases and was analyzed for their drug-likeliness properties and chemical classification. Further, the information of protein molecules targeted by phytochemicals was collected and poly-pharmacological action of each was assessed based on the number of protein targets that a phytochemical may target. Functional significance of protein targets was assessed using the pathway and module associations. The high confidence phytochemical - protein target pairs involved in this disorder were identified and their molecular interactions were assessed on the basis of *in-silico* docking studies. The complete methodology of this study is presented in Fig. 1.

## Material and Methods

### Identification of anti-epileptic herbs (AEHs) and drugs (AEDs)

To collect the information of the anti-EP herbs prescribed in Ayurveda system of medicine, pertinent research articles in PubMed-NCBI (https://www.ncbi.nlm.nih.gov/pubmed/) were shortlisted and manually inspected. For the identification of the AEDs that are in current use and also under clinical trials, DrugBank database (https://www.drugbank.ca/)^20^ screening and literature survey were performed^36,37^.

### Phytochemical dataset preparation of anti-epileptic herbs

A list of phytochemicals present in the identified Anti-epileptic herbs was compiled from 3 database sources: Duke’s phytochemical database (https://phytochem.nal.usda.gov/phytochem/search), TCMSP (Traditional Chinese Medicine Systems Pharmacology)^38^, and PCIDB (PhytoChemical Interactions DB) (https://www.genome.jp/db/pcidb). PubChem (https://pubchem.ncbi.nlm.nih.gov/) and ChEMBL (https://www.ebi.ac.uk/chembl/) databases of chemical compounds were used to derive the chemical information of phytochemicals. 2D and 3D chemical structures of phytochemicals were obtained using OpenBabel 2.4.1 software^39^. The phytochemicals, for which no chemical information could be obtained, were not considered in this study.

### Protein target identification of AEDs and phytochemicals in AEHs (phytochemical dataset)

The identification of potential drug targets is considered a key step towards the drug development procedure^40,41^. Here, three target prediction softwares were used to collect the information of potential protein targets of the phytochemical dataset. (1) BindingDB (https://www.bindingdb.org/), a web-accessible public database that contains the protein interaction information of the potential drug targets with their ligand molecules^19^. For accessing the high confidence interaction pairs, BindingDB was searched with interaction score of ≥0.85. (2) STITCH 5.0 (http://stitch.embl.de/), which catalogs the information of manually curated as well as experimentally validated protein-chemical interactions^18^. Only the chemical interactions with STITCH confidence score of ≥0.4 were considered. (3) Swiss Target Prediction (http://www.swisstargetprediction.ch/), a web server for the target identification of bioactive small molecules that uses the combination of 2D and 3D similarity measures for the prediction of top-15 targets^16^. For all the predictions, search was limited to “*Homo sapiens*”.

### EP targets (EP-gene pool)

The data of EP associated genes were collected from three databases: (1) EpilepsyGene (http://www.wzgenomics.cn/EpilepsyGene/), a genetic resource which accounts for the information on epilepsy-related genes and mutations, collected from various research publications^42^; (2) OMIM database (http://omim.org/), a frequently updated catalogue of human genes, genetic phenotypes and traits for establishing a relationship between phenotype and genotype; and (3) DisGeNET, a collection of disease-associated genes and variants associated with *Homo sapien*s^43^. A list of 1,179 non-redundant genes corresponding to epilepsy is developed from these sources and is detailed in Supplementary File 1, Table S1.

### Compound classification and clustering

The chemical classification of phytochemicals was obtained using “Classyfire”. The fully automated tool of the Classyfire utilizes the information of chemical structures to assign a chemical class to the query compound with hierarchical classification including the information of kingdom, class, super class etc. This provides the hierarchical classification of chemical compounds based on the chemical ontology of approx. 4,825 organic and inorganic compounds^44^. Online chemical cluster service provided by ChemMine tools^45^ was used for the clustering of phytochemicals. For the clustering of compounds based on structural similarity, firstly atom pair descriptors (*i.e*. features) of each subjected chemical compound were generated by the underlying algorithm. Using the set of common and unique features, Tanimoto coefficients (described in detail in the following subsection “Similarity index calculation”) were calculated and a similarity matrix was created. The generated similarity matrix was utilized for the clustering of phytochemicals and the results were presented in the Newick tree format. Results of cluster analysis with those of chemical information obtained from Classyfire server were integrated to display the complete information of phytochemicals.

### Pharmacokinetic prediction and drug-likeliness evaluation

Estimation of pharmacological properties of small molecules is considered a crucial step towards the drug discovery approaches. In that direction, owing to the disadvantage of *in vivo* estimation as time consuming and expensive, *in silico* methods have become inevitable approaches. In this study, we used a graph-based signature method namely, pkCSM for the prediction of pharmacokinetic and toxicity properties of the compounds^46^. Four absorption, distribution, metabolism, excretion and toxicity (ADMET) related parameters including Lipinski “rule of five” criterion, Caco-2 permeability, intestinal absorption and toxicity assessment were used to screen drug-like molecules from the list. Lipinski’s “rule of five” criterion is considered as a basis for estimating the drug like nature of the chemical compounds. According to its five component measure, a chemical compound must have a number of H-bond acceptors less than 10, number of H-bond donors less than 5, molecular weight less than 500 Dalton and logP value of less than 5 for its proper absorption and permeability^47^. Permeability coefficient across the human colon carcinoma cell lines Caco-2 is considered as an important parameter for predicting the absorption of orally administered drugs^48^, we used the threshold of Caco-2 permeability score > 0.9 and intestinal absorption value of >30% as a cut-off. Lastly, phytochemicals negative for Hepatotoxicity and Ames toxicity were selected. Only phytochemicals passing these above criteria were selected for subsequent analysis and declared as putative drug-like molecules. The ADMET (Absorption, Distribution, Metabolism, Excretion and Toxicity) properties of 349 putative drugable phytochemicals referred to as DPCs are provided in Supplementary File 2, Table S2.

### Similarity index calculation

For evaluating the similarity between 349 DPCs and drugs available at DrugBank, Tanimoto coefficient (TC) was calculated using OpenBabel (version 2.4.1)^39^. A path-based fingerprint which indexes small molecule fragments upto the length of seven atoms *i.e*. FP2 was used for the TC calculation. TC between chemical compound C_1_ and C_2_ is given by,

$$T{C}_{(C1,C2)}=\frac{{N}_{({C}_{1},{C}_{2})}}{{N}_{({C}_{1})}+{N}_{({C}_{2})}-{N}_{({C}_{1},{C}_{2})}}$$

where, ${N}_{({C}_{1})}$ and ${N}_{({C}_{2})}$ referes to the number of molecular fingerprints present in C_1_ and C_2_ respectively^49,50^. While ${N}_{({C}_{1},{C}_{2})}$ represents number of molecular fingerprints common to both C_1_ and C_2_. Range of the TC varies from 0–1, where 0 represents the minimum and 1 represents the maximum similarity.

Only the drugs corresponding to “drugbank_approved_structure” data, present in DrugBank dataset library were selected for the comparison. Tanimoto score calculated for each pair of 349 DPCs with 2,159 drugs (of 2,337 drugs considered in this study) are given in the Supplementary File 3, Table S3.

### Network analysis

The PPI (protein-protein-interaction) data of protein targets was fished from STRING, with search limited to “*Homo sapiens*” and a score ≥0.9 corresponding to high confidence interaction pairs^51^. Cytoscape (version 3.0)^52^, an open source software was used for the network construction, visualization and analysis. Network construction and analyses were performed for the following cases: (1) Herb-Phytochemical (H-PC) network, (2) Drugable phytochemical-Protein target (DPC-PT) network, (3) Protein target-Human pathway (PT-HP) network, and (4) Epilepsy protein-protein interaction (EP-PPI) network. The pathway database of Kyoto Encyclopedia of Genes and Genomes (KEGG), (http://www.genome.jp/kegg/pathway.html) was used to retrieve the information of human pathways associated with protein targets^53^. Molecular COmplex DEtection (MCODE) algorithm was opted to identify and organize the functional modules in the EP-PPI network. These densely connected regions *i.e*. modules are known to possess network like properties and tend to be composed of a set of proteins that in coordination with each other regulate specific functions^54^. Clusters were analyzed for the gene ontology enrichment using DAVID Functional Annotation Bioinformatics Microarray Analysis v6.8^55^.

### Screening of NeuMoDs (Neuro Modulatory DPCs)

In order to identify DPCs capable of providing similar or better neuromodulatory potential with respect to DrugBank compounds, we developed a comprehensive methodology that consists of three main steps:

(A) **Neuromodulatory Proteins (NM-Proteins) dataset:** A list of all the protein targets of DPCs that are involved in 10 KEGG pathways associated with nervous system and are also available in the “approved protein target list” of DrugBank.
(B) **Compound datasets: (i) D-dataset:** All the DrugBank molecules known to target one or more protein-targets of identified DPCs.
  **(ii) D1-dataset:** DPCs that have been predicted to target the NM-Proteins.
  **(iii) D2-dataset:** DrugBank molecules reported to target the NM-proteins.
(C) **Selection criteria:** To select the desired DPCs, both the following two conditions should be satisfied.
  (i) Tanimoto coefficient (TC) between a DPC in D1-dataset and DrugBank molecule in D-dataset >0.85.
  (ii) Tanimoto coefficient (TC) between a DPC in D1-dataset and DrugBank molecule in D-dataset ≠ 1. Also, their simplified molecular input line entry system (SMILES) should not be identical.

SMILES is a chemical notation system designed for modern chemical information processing. Based on the molecular graph theory principles, SMILES allows the meticulous specification of the chemical structure in a linear string notation, as of natural grammar, enabling the chemists to draw and describe the 2D valence-oriented picture of the chemical molecule under study^39^.

A tripartite network was constructed in which the middle layer was of NM-proteins and interactions with DPCs passing this selection criterion were on one side and DrugBank molecules of D2-dataset were on the other side. A systematic analysis of this network was performed that include highlighting high-confidence interactions and multi-targeting DPCs to report the novel NeuMoDs.

### Drug-target interaction validation and visualisation

Energy minimization, molecular docking and binding energy calculations were performed to validate the potential drug-target interactions. For this, the crystal structures of protein targets were obtained from RCSB Protein Data Bank (PDB) (www.rcsb.org)^56^. The proteins for which no crystal structure is available in PDB was modeled using Phyre2^57^. The refinement of the modeled structures was performed using MOdrefiner^58^ and GROningen MAchine for Chemical Simulation (GROMACS) v5.0 package (http://www.gromacs.org/) was used for the energy minimisation using Gromos96 53a6 force field using steepest descent method.

Non-covalent interactions between the proteins and ligands were assessed using molecular docking studies, for that AutoDock v4.2^59^ and AutoDock Vina packages^60^ were used. Ligplot^+^, a graphical system that utilises 3D coordinates to generate the interaction maps was used to visualize the interaction present in protein-ligand complex^61^.

## Results and Discussion

### Identification of anti-epileptic herbs (AEHs) and anti-epileptic drugs (AEDs)

We could identify 63 AEHs *via* an extensive review of the PubMed research articles and assigned a unique identifier to each herb. The detailed information of the identified herbs including their scientific name, the unique identifier and the reference source is presented in Table 1. A list of 40 AEDs currently used for the management of epilepsy or under clinical trials was also enlisted. The chemical information of these drugs including their DrugBank Id, PubChem Id and SMILES notation is given in Supplementary File 4, Table S4.

**Table 1:** List of anti-epileptic herbs (AEHs) used in this study.

S. No.	Herb-ID	Scientific name of herb	Reference
1	EP1	*Acorus calamus*	^104^
2	EP2	*Albizia lebbeck*	^105^
3	EP3	*Aloe vera*	^106^
4	EP4	*Anacyclus pyrethrum*	^107, 108^
5	EP5	*Anethum graveolens*	^109– 111^
6	EP6	*Anisomeles malabarica*	^112^
7	EP7	*Anthocephalus cadamba*	^113^
8	EP8	*Antiaris toxicaria*	^114, 115^
9	EP9	*Argyreia speciosa*	^116^
10	EP10	*Asparagus racemosus*	^117^
11	EP11	*Bacopa monnieri*	^118– 121^
12	EP12	*Bixa orellana*	^122, 123^
13	EP13	*Boerhavia diffusa*	^124^
14	EP14	*Brassica nigra*	^125^
15	EP15	*Bryophyllum pinnatum*	^126, 127^
16	EP16	*Butea monosperma*	^105, 128^
17	EP17	*Caesalpinia sappan*	^129^
18	EP18	*Calotropis gigantean*	^130^
19	EP19	*Calotropis procera*	^131, 132^
20	EP20	*Carum copticum*	^133^
21	EP21	*Cedrus deodara*	^134^
22	EP22	*Centella asiatica*	^135– 137^
23	EP23	*Cicer arietinum*	^138^
24	EP24	*Cissus quadrangularis*	^139^
25	EP25	*Clitorea ternatea*	^140^
26	EP26	*Croton tiglium*	^141^
27	EP27	*Curcuma longa*	^69, 142, 143^
28	EP28	*Cyperus rotundus*	^144^
29	EP29	*Delphinium denudatum*	^145– 147^
30	EP30	*Emblica officinalis*	^148, 149^
31	EP31	*Ferula asafoetida*	^150^
32	EP32	*Ficus carica*	^151^
33	EP33	*Glycyrrhiza glabra*	^152– 156^
34	EP34	*Hibiscus rosa-sinensis*	^105^
35	EP35	*Hyoscyamus niger*	^157^
36	EP36	*Indigofera tinctoria*	^158^
37	EP37	*Mimosa pudica*	^159^
38	EP38	*Moringa oleifera*	^160^
39	EP39	*Myristica fragrans*	^161^
40	EP40	*Nardostachys jatamansi*	^162^
41	EP41	*Nigella sativa*	^163– 167^
42	EP42	*Ocimum gratissimum*	^168, 169^
43	EP43	*Ocimum sanctum*	^170^
44	EP44	*Passiflora incarnate*	^171, 172^
45	EP45	*Pinus roxburghii*	^173^
46	EP46	*Portulaca oleracea*	^174^
47	EP47	*Punica granatum*	^175^
48	EP48	*Ricinus communis*	^176^
49	EP49	*Rubia cordifolia*	^177^
50	EP50	*Sesbania grandiflora*	^178^
51	EP51	*Smilax china*	^179^
52	EP52	*Solanum nigrum*	^180^
53	EP53	*Trichosanthes tricuspidata*	^181^
54	EP54	*Vetiveria zizanioides*	^182^
55	EP55	*Vitex negundo*	^156, 183^
56	EP56	*Withania somnifera*	^184, 185^
57	EP57	*Zingiber officinale*	^186, 187^
58	EP58	*Zizyphus jujuba*	^188, 189^
59	EP59	*Piper longum*	^190, 191^
60	EP60	*Terminalia chebula*	^192^
61	EP61	*Pimpinella anesum*	^193^
62	EP62	*Ruta graveolans*	^194^
63	EP63	*Datura stramonium*	^195^

### Association network of phytochemicals with AEHs and their chemical classification

**Figure 2:**
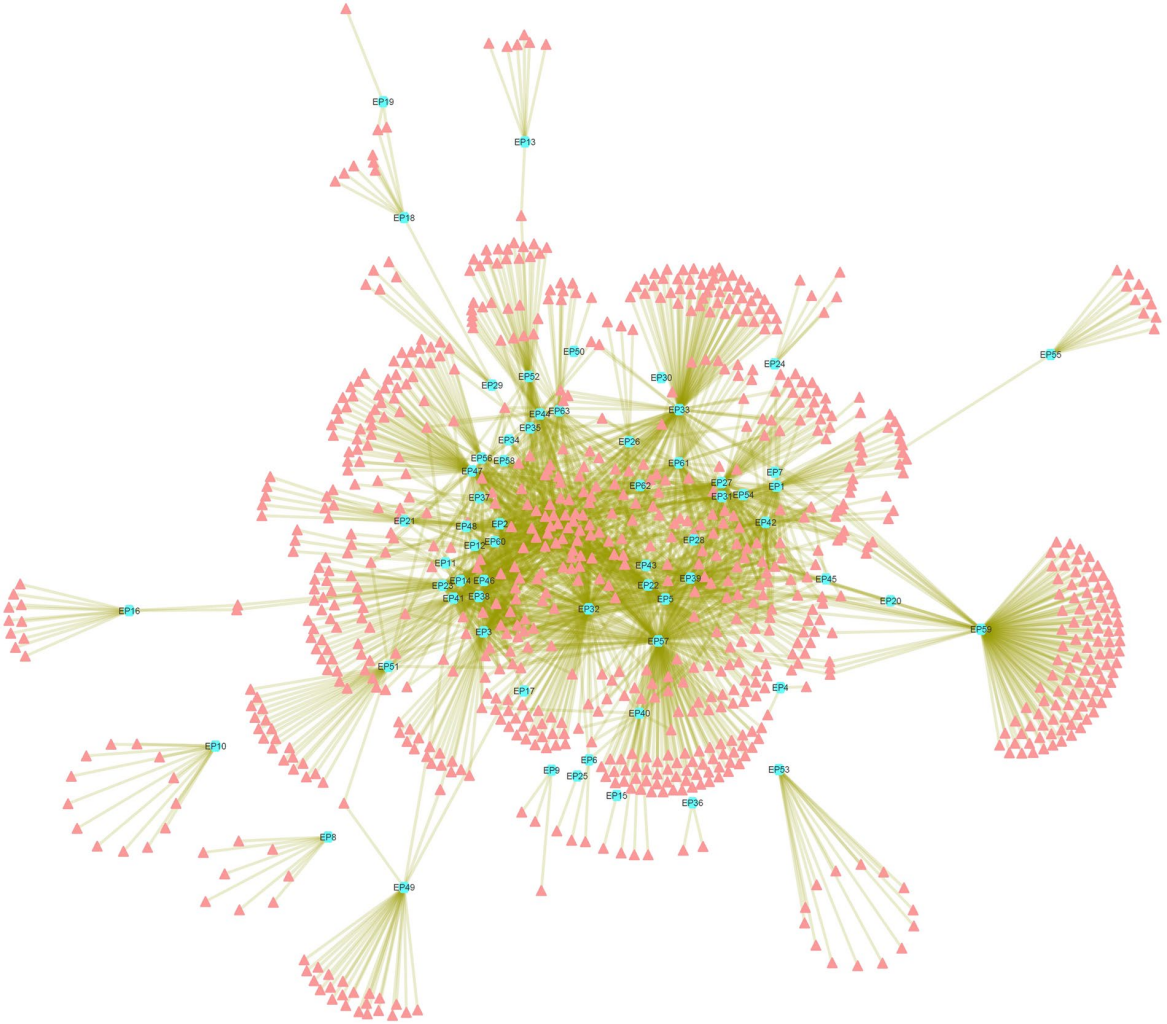
Herb-Phytochemical (H-PC) Network: The H-PC network represents associations of 867 unique phytochemicals (peach coloured nodes) with 63 anti-EP herbs (Cyan nodes). Herb *“Zingiber officinale”* (EP57) is found to have maximum number of phytochemicals among all the anti-EP herbs, followed by “*Piper longum”* (EP50), “*Anethum graveolens”* (EP5) and “*Glycyrrhiza glabra”* (EP33).

The phytochemical (PC) dataset of 63 anti-epileptic herbs consists of 867 unique entries out of total 1,993 PCs collected. For the study, each phytochemical of the PC dataset is assigned a Phytochemical-ID (Supplementary File 5, Table S5). Detailed mapping of these 867 PCs to their corresponding herb is presented in Supplementary File 6, Table S6. This information was used as an input to construct the herb-phytochemical (H-PC) interaction network (Fig. 2). The network analysis shows that several PCs are shared by many of the anti-EP herbs while others are specific in nature. PC157 (palmitic acid) is the most common PC as it is found in 29 out of 63 anti-EP herbs. Other PCs enriched in the AEHs are PC350 (ascorbic acid), PC170 (oleic-acid), PC044 (linoleic-acid) with the degree centrality (C_d_) value of 28, 27 and 25 respectively. PC350 (*i.e*. ascorbic acid) is one of the most widely studied PC having implications in EP^62^. Deficiency of PC350 is known to contribute in increasing the severity of the PTZ induced seizures^63^. Out of 867 phytochemicals, 349 were found to have drug-likeliness properties (using the methods described in the subsection “Pharmacokinetic prediction and drug-likeliness evaluation”) and among these drugable PCs (DPCs); PC703(phosphorus), PC158 (pyridine-3-carboxylic acid) are shared by 24 and 18 anti-EP herbs, respectively. Herb *“Zingiber officinale”* (EP57) is found to have a maximum number of DPCs *i.e*. 104.

**Figure 3:**
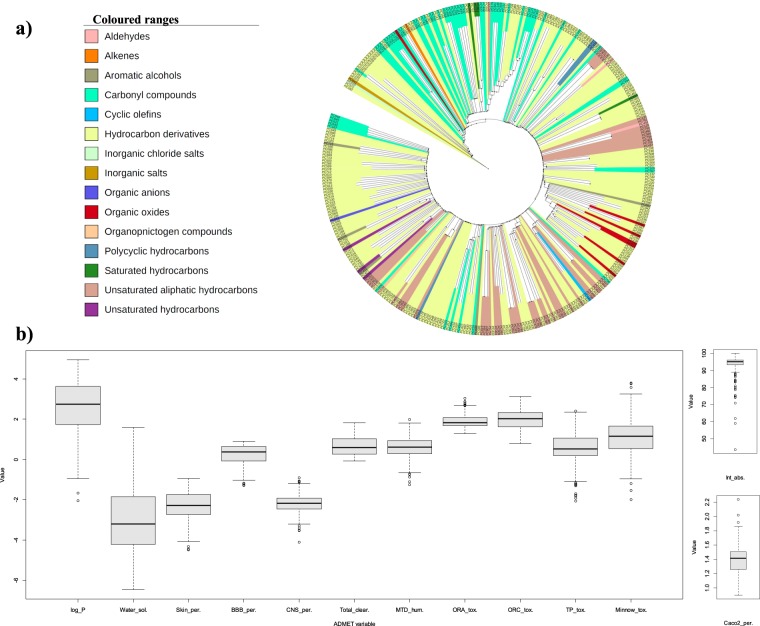
(**a**) Clustering and chemical classification of DPCs: The hierarchical clustering of 348 DPCs based on their atom-pair descriptors and Tanimoto coefficient, obtained using Chemmine tool. One DPC corresponding to the PC703 with the chemical class of “Non-metal compounds” could not be clustered and therefore not represented in this tree layout. (**b**) ADMET properties of DPCs: Box and whisker plot represents the 13 ADMET properties of the 349 DPCs. The properties were evaluated using the pkCSM server and are as follows; octanal-water partition coefficient (log_P), water solubility (Water_sol.), skin permeability (Skin_per.), blood-brain permeability (BBB_per.), central nervous system permeability (CNS_per.), total clearance (Total_clear.), maximum tolerated dose-humans (MTD_hum.), oral rat acute toxicity (ORA_tox.), oral rat chronic toxicity (ORC_tox.), *T. Pyriformis* toxicity (TP_tox.), minnow toxicity (Minnow_tox.), caco-2 cell permeability (Caco-2_per.) and intestinal absorption (Int_abs.).

The comprehensive organization of 349 drugable phytochemicals (DPCs), obtained from drugability analysis is found to be distributed in the 16 broad chemical classes (Fig. 3a). Detailed classification of the DPCs reveals that the class corresponding to terpenoids especially “sesquiterpenoids” is highly prevalent in this dataset. This observation may be due to the fact that terpenoids constitute the most diverse and largest class of natural compounds^64,65^. Together with their involvement in plant growth and survival, both primary and secondary metabolites of the terpenoid biosynthetic pathway possess commercial, ecological as well as medicinal significance^64^. Moreover, the dataset is highly enriched for the compounds having the capabilities of crossing the blood-brain barrier viz. hydrocarbons, terpenes and carbonyl compounds^66^. Various studies have correlated the anticonvulsant activity of plant-based products with their terpenoids composition^67,68^. The anticonvulsant properties of sesquiterpenoid metabolites have also been previously identified in zebrafish and mouse seizure models^69,70^. This shows that these compounds may be given further attention for detailed examination as they provide reliable data for the future anti-epileptic drug discovery approaches.

Other than the class of terpenoids, compounds belonging to “Methoxyphenols” are enriched in this dataset. Although the use of phenolic compounds in reference to epilepsy is less explored, our findings suggest that the detailed investigation of such compounds could be of considerable importance. The chemical class of each DPC can be checked in Supplementary File 7, Table S7.

### Drugable phytochemical - Protein target (DPC-PT) network

To understand the molecular interactions of DPCs with human proteins, a bipartite DPC-PT network was constructed (Supplementary Fig. 1). This network consisted of the mapping of DPCs (349) with their protein targets (4,982) that are obtained using three algorithms of target prediction (described in Materials and Methods). Prioritization of each 16,329 phytochemical-protein pairs was performed on the basis of their prediction from each of the three target prediction algorithms. Sixty-six (66) high confidence DPC-PT pairs were identified as these were predicted from all the three target prediction algorithms used (Supplementary File 8, Table S8).

**Figure 4:**
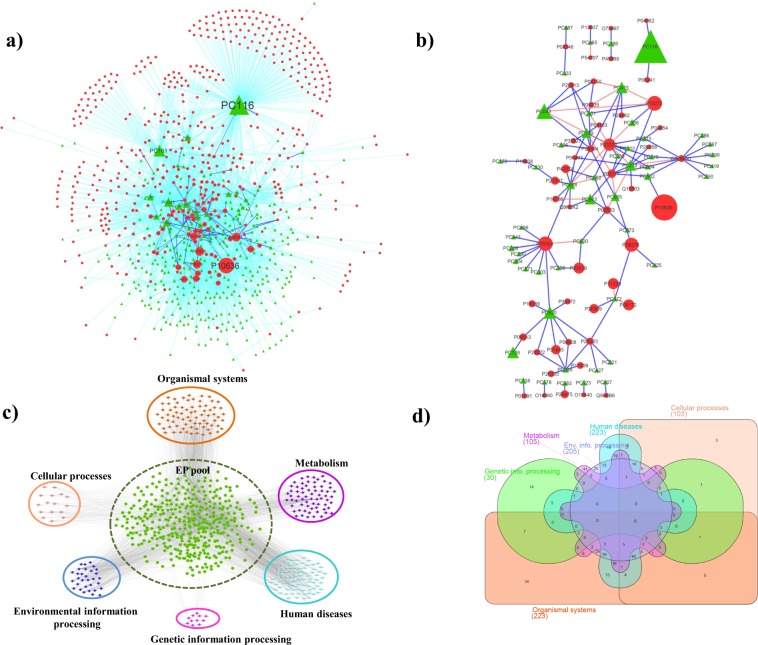
(**a**) A sub-network of DPC-PT network specific to Epilepsy (DPC-PTE): This network consists of 838 nodes (336- drugable phytochemicals; 502 proteins) and 3,002 DPC-PT pairs, specific to the EP-pool proteins. Phytochemicals are represented by green coloured nodes and EP-pool proteins with red coloured nodes. Interactions among the phytochemical-protein pairs are either represented as cyan (predicted by any 1 protein target algorithms), blue (predicted by any 2 protein target algorithms) or orange coloured edges (predicted by all 3 protein target algorithms). Size of the nodes is based on their corresponding degree value in the network. Among phytochemicals, PC116 holds the maximum degree value. **(b**) A high confidence sub-network of DPC-PTE network: For specifically examining the high-confidence interactions among the DPCs and protein targets associated with EP, a sub-network of DPC-PTE network is constructed by considering the interactions predicted by either 2 or 3 target prediction algorithms. **(c**) PTE-HP (Protein targets associated with Epilepsy - Human pathway) Network: The pathway enrichment network consists of 677 nodes and 2,862 edges, specific to the EP-pool proteins. 400 proteins of EP-pool targeted by any of the DPCs are found to be involved in the 6 broad KEGG pathway classes *i.e*. Organismal systems, Cellular processes, Environmental information processing, Genetic information processing, Human diseases and Metabolism. The 6 main pathway classes are arranged around the proteins of EP-pool, represented in green coloured nodes in the center of the network. **(d**) Distribution of EP-pool proteins among KEGG pathway classes: Venn-diagram representing the distribution of 400 proteins of the PTE-HP network among the 6 broad KEGG pathway classes. The class corresponding to the “Organismal systems” and “Human diseases” are found to be highly enriched with EP-proteins while the class of “Genetic information processing” includes the least number of proteins *i.e*. 30. Many proteins are shared among these classes, the feature attributed towards association of a single protein in multiple pathways.

A sub-network of DPC-PT network [referred as DPC-PTE; drugable phytochemical-protein targets associated with epilepsy] (Fig. 4a) consisting of 838 nodes (336-phytochemicals; 502 proteins) and 3,002 DPC-PT pairs, specific to the EP-gene pool (mentioned in material and methods section) was derived to highlight the interactions among epilepsy proteins and DPCs that may target them. In this network, three DPCs corresponding to PC116 (glycerol), PC161 (ethanol) and PC043 (17-beta-estradiol) possess the target degree centrality value (C_d_) more than 100 *viz*. 259, 140 and 102 respectively. This indicates that these phytochemicals may play a key role in regulating the EP targets *via* regulating multiple proteins simultaneously. From the protein target list, P10636 (C_d_ = 95), P10275 (C_d_ = 95), P35354 (C_d_ = 91) retain the highest number of contacts with the DPCs in DPC-PT network. P10636 is a MAPT (microtubule-associated protein tau) and its involvement in various neurological diseases is well known^71^. The microtubule overexpression has been linked to the temporal lobe epilepsy *via* collateral sprouting of hippocampal mossy fibers^72^. Also, various AEDs (Anti-epileptic drugs) are shown to induce changes in the expression of androgen receptors P10275, when taken by patients with temporal lobe epilepsy^73^. Expression of protein P35354 (prostaglandin G/H synthase-2) has been shown to have an elevated induction upon seizures^74^. To access high confidence interactions of DPC-PTE network, a sub-network corresponding to compound-protein interactions was derived by including only those pairs that were returned by at least two of the three algorithms used for target prediction (Fig. 4b). Detailed inquiry of this sub-network helped us to identify the prospective role of apigenin (PC668) in the regulation, based on its protein-targeting capability (C_d_ = 9), compared to other DPCs. Among the total 29 DPCs of 66 high confidence DPC-PTE pairs, maximum contribution of 10 from EP33 (*Glycyrrhiza glabra*) and 9 from EP47 (*Punica granatum*) shows that anti-epileptic action of these herbs is worthy of attention.

### Protein Target-Human pathway (PT-HP) network

PT-HP network illustrates the association of protein targets (protein targets of DPCs) with human pathways based on KEGG analysis. Detailed mapping of all the 4,982 DPC’s targeted proteins into 314 human pathways is presented in Supplementary File 9, Table S9. Only 3,030 proteins out of 4,982 DPC’s targeted protein targets show their involvement in the human pathways, according to KEGG analysis. This data was used as an input to construct a bipartite PT-HP network, consisting of 3,344 nodes (3,030 protein targets; 314 human pathways) and 15,535 edges. Proteins involved in epilepsy were selected from PT-HP network, and a sub-network specific to epileptic proteins and their regulatory pathways was constructed (PTE-HP, Protein Targets of Epilepsy-Human pathway) (Fig. 4c). PTE-HP consisting of 677 nodes and 2,862 edges was analyzed for the human pathway enrichment of the EP proteins. This is essential to detect the disease-associated pathways and to understand the underlying mechanism of “protein-disease associations” of epilepsy in detail. Also, the analysis could help in the identification of novel pathways and their regulatory proteins in the context of epilepsy. As shown in Fig. 4c, it is observed that proteins involved in pathways associated with “organismal systems” and “human diseases” have the highest enrichment in this dataset followed by the categories belonging to the classes of “environment processing” and “metabolism”. Many proteins were shared among these classes, a feature attributed towards association of a single protein in multiple pathways. A set of 9 proteins corresponding to P29475 (C_d_ = 11), O60503 (C_d_ = 43), P31749 (C_d_ = 76), Q9NQ66 (C_d_ = 46), P19174 (C_d_ = 33), P16885 (C_d_ = 31), Q13393 (C_d_ = 14), O14939 (C_d_ = 12) and P35354 (C_d_ = 16) were highlighted in this step, as they are shown to possess multi-level regulatory behaviour *via* its involvement in each of these four major pathway classes. Such proteins may be considered as important targets for the EP because modulation at these levels will give a direct effect on the pathways of their involvement. Distribution of EP proteins among the six major pathway classes is shown in Fig. 4d in the form of the Venn-diagram constructed using InteractiVenn (http://www.interactivenn.net/)^75^.

Among the class of “organismal systems”, the pathway corresponding to “path: hsa04724; glutamatergic synapse” share the maximum number of proteins. Regulation of glutamate homeostasis plays a key role in the pathophysiology of epilepsy^76^ and altered behavior of this pathway upon the episode of neonatal seizures has also been reported^77^. Also, the “path: hsa01100” (metabolic pathways) and “path: hsa04080” (Neuroactive-ligand-receptor interaction pathway) have maximum number of EP proteins involved in them. These findings support previous studies according to which there exists a therapeutic relationship between brain activity and metabolism to treat neurological disorders^78,79^. Although, direct correlation of the “path: hsa04080” and epilepsy could not be established, this pathway has remained as an important source for the development of various therapeutic strategies against various neurological disorders^80^. Thus, it may be correlated that regulation of genes corresponding to this neurological pathway may be responsible for the wide spectrum alteration in various neurological functions in epilepsy. Detailed information of human pathways with the number of EP pool proteins associated with them, is presented in Supplementary File 10, Table S10.

In the following, we detail our network analysis for some specific case studies.

#### Case study-I: Investigation of the neuromodulatory effects of AEHs

To explore the neuromodulatory potential of AEHs, we attempted to uncover the underlying mechanism of neuromodulation by highlighting the potential DPC-PT interactions potentially responsible for their regulation. Towards this end, pathways specific to nervous system and protein targets involved in these pathways were selected for further analysis. A sub-network specific to all the 10 KEGG pathways associated with nervous system namely, glutamatergic synapse (path:hsa04724), GABAergic synapse (path:hsa04727), cholinergic synapse (path:hsa04725), dopaminergic synapse (path:hsa04728), serotonergic synapse (path:hsa04726), long-term potentiation (path:hsa04720), long-term depression (path:hsa04730), retrograde endocannabinoid signaling (path:hsa04723), synaptic vesicle cycle (path:hsa04721) and neurotrophin signaling pathway (path:hsa04722) and their associated proteins was focused. This sub-network consisting of 110 nodes (100 proteins; 10 pathways) and 200 edges was analysed in detail for the following sub-cases (Supplementary Fig. 2 and Supplementary File 11, Table S11):

#### Ia: Identification of regulatory DPCs for metabotropic glutamate receptors (mGluRs)

**Figure 5:**
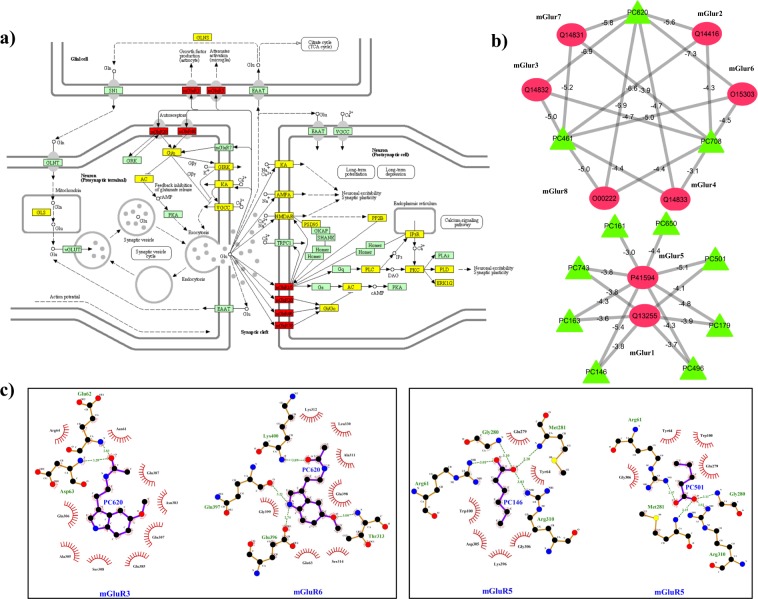
(**a**) Glutamatergic synapse (path:hsa04724) pathway mapping of the protein targets of DPCs: The location of protein targets of DPCs in the KEGG pathway hsa04724 is represented in yellow and red coloured boxes, red ones are specific to mGluRs. Reprinted with permission from Kyoto Encyclopedia of Genes and Genome, https://www.kegg.jp/kegg/kegg1.html. **(b**) Sub-network of mGluRs and their regulatory DPCs: Green coloured nodes of the network correspond to 11 DPCs (PC146, PC161, PC163, PC179, PC461, PC496, PC501, PC620, PC650, PC708 and PC743) that possess the tendency to regulate 8 classes of mGluRs (Red coloured circular nodes). The binding energy values (kcal/mol) of the mGluRs and their regulatory DPCs are represented along with their corresponding edges in the network. For docking studies, the structure of mGluR6, mGluR4 and mGluR8 were modelled using PHYRE2 while for others they were obtained from RCSB-PDB with following PDB-IDs: 3KS9 (mGluR1), 4XAS (mGluR2), 6B7H (mGluR3), 3LMK (mGluR5) and 3MQ4 (mGluR7). (**c**) The interaction analysis of the mGluRs and their regulatory DPCs: Docked complexes with the best binding energy values are analysed for the hydrophobic interactions as well as hydrogen bonded residues using Ligplot^+^. The ligplots enclosed in boxes are the representative cases of multi-targeting and synergistic actions of selected phytochemicals respectively. Protein residues represented along the arcs are involved in the hydrophobic interactions whereas the residues involved in the hydrogen bonding are represented using dashed lines. The PC620 is shown to possess much negative binding energy compared to the other 10 DPCs and the interaction analysis highlights the importance of the acetamide group (-NHCOCH_3_) in the interaction.

The pathway relationship of above sub-network shows that the glutamatergic synapse (path:hsa04724) is associated with 32% protein targets and retrograde endocannabinoid signaling (path:hsa04723) is associated with 27% protein targets. Mapping of the protein targets in the glutamatergic synapse pathway (path:hsa04724) is shown in Fig. 5a. As already discussed about the role of Glutamatergic mechanism in the condition of seizures and epilepsy, detailed literature data could link up the role of metabotropic glutamate receptors (mGluRs) for inducing epileptic symptoms in the patients^81,82^. Also, many of the current antiseizure drug development strategies are focusing on the compounds that have potential to modulate the glutamatergic signaling *via* mGluRs^81^. For the identification of such potential drug candidates from our dataset of AEHs, proteins corresponding to mGluRs were backmapped in the DPC-PT network. This led to the selection of 11 DPCs (from D1 dataset) corresponding to (PC146; caproic acid), (PC161; ethanol), (PC163; lauric acid), (PC179; butyric acid), (PC461; camphor), (PC496; propionic acid), (PC501; valeric acid), (PC620; melatonin), (PC650; benzoic acid), (PC708; lactic acid) and (PC743; 2-nonynoic acid). A sub-network corresponding to the interactions between these 11 DPCs and 8 proteins (corresponding to mGluRs), presented in Fig. 5b was evaluated for examining the multi-targeting nature of these DPCs. We found that PC461, PC708 and PC620 target 6 out of 8 mGluRs. Docking studies were carried out for each of these interaction pairs and binding free energy values were also calculated that are mentioned as the edge weights in the network shown in Fig. 5b. The best-docked conformation was selected based on the binding energy value, *i.e*. one with lowest binding energy and was used to represent the molecular interactions via Ligplots. All of these DPCs are found to possess a very good binding affinity to mGLuRs with binding energy values varying in the range of −3.1 to −7.3 kcal/mol. The best binding energies were observed for PC620 with mGluR3, mGluR6 and mGluR8. The 2D representation of the interactions shows that the targets of PC620 are involved in multiple hydrogen bonds and hydrophobic contacts (Fig. 5c).

Further mGluR5 (P41594) protein was checked for its regulatory phytochemicals and 8 DPCs (PC146, PC161, PC163, PC179, PC496, PC501, PC650 and PC743) could be selected in this regard (see Fig. 5b). We selected this protein for the detailed analysis because mGluR5 antagonists have been reported to be effective against seizure patients^83,84^. Such antagonists may be looked upon to control the seizures in case of epilepsy also. The above mentioned 8 DPCs were mapped in H-PC network to identify the AEHs containing them. Mapping of 5 of these 8 DPCs in EP-42 (*Ocimum gratissimum*) and 3 in both EP-33 (*Glycyrrhiza glabra*) and EP-26 (*Croton tiglium*) reflect their mGluR5 regulatory behavior. It was interesting to note that the selected 8 regulators of mGluR5 also show their affinity towards mGluR1. The binding affinity of each of the 8 DPCs with mGluR5 (P41594) and mGluR1 (Q12355) are represented along the edges of the network shown in Fig. 5b. Detailed examination of the interaction pairs and their associated binding affinity draws attention towards the multi-targeting and synergistic behavior of DPCs (see Fig. 5b). While PC620 is shown to hold a multi-targeting property for mGluR3 and mGluR6 at a good binding affinity score, synergistic action of PC146 and PC501 to regulate mGluR5 is of considerable importance. The interaction analysis also supports the multi-targeting and synergistic action of DPCs (Fig. 5c). The data might be investigated in detail, for the screening of lead molecules that may be found effective for their anti-epileptic actions.

#### Ib: Screening of novel neuromodulatory DPCs (NeuMoDs)

Towards prioritization of DPCs obtained from AEHs against the above mentioned 100 proteins involved in neuromodulatory pathways, a comprehensive methodology was developed as detailed in the Methods Section 2.9. In the following, the results obtained in various steps are described and discussed in detail.

Firstly, we mapped the 100 proteins obtained in the previous section to ‘approved protein target list’ comprising of 2,669 proteins as provided by DrugBank. 81 proteins could be successfully mapped in this approved list and the further study was restricted to the set of these 81 neuromodulatory proteins only (NM-proteins). Secondly, for exploring the potential of DPCs in regulating the NM-proteins, these proteins were backmapped to the DPC-PT network and DrugBank database. From the DPC-PT network, 241 DPCs were found to have a role in the regulation of NM-proteins. From the DrugBank database, 1,684 drugs (of total 2,337 approved drugs listed in DrugBank, D dataset) were found to target 1,311 proteins (of 4,982 PTs of all DPCs). Out of these, a total of 467 drugs were found to be associated with the selected 81 NM-proteins. In this manner the regulatory molecules of the selected 81 NM-proteins were obtained from two facets, one which includes the DPCs of anti-EP herbs (241 DPCs, D1 dataset) and the second includes drugs given in DrugBank database (467 drugs, D2 dataset). The drugs and DPCs of D, D1 and D2 datasets with their associated proteins are given in Supplementary File 11, Table S11 and details of 2,669 ‘approved protein target list’ is given in Supplementary File 3, Table S3.

**Figure 6:**
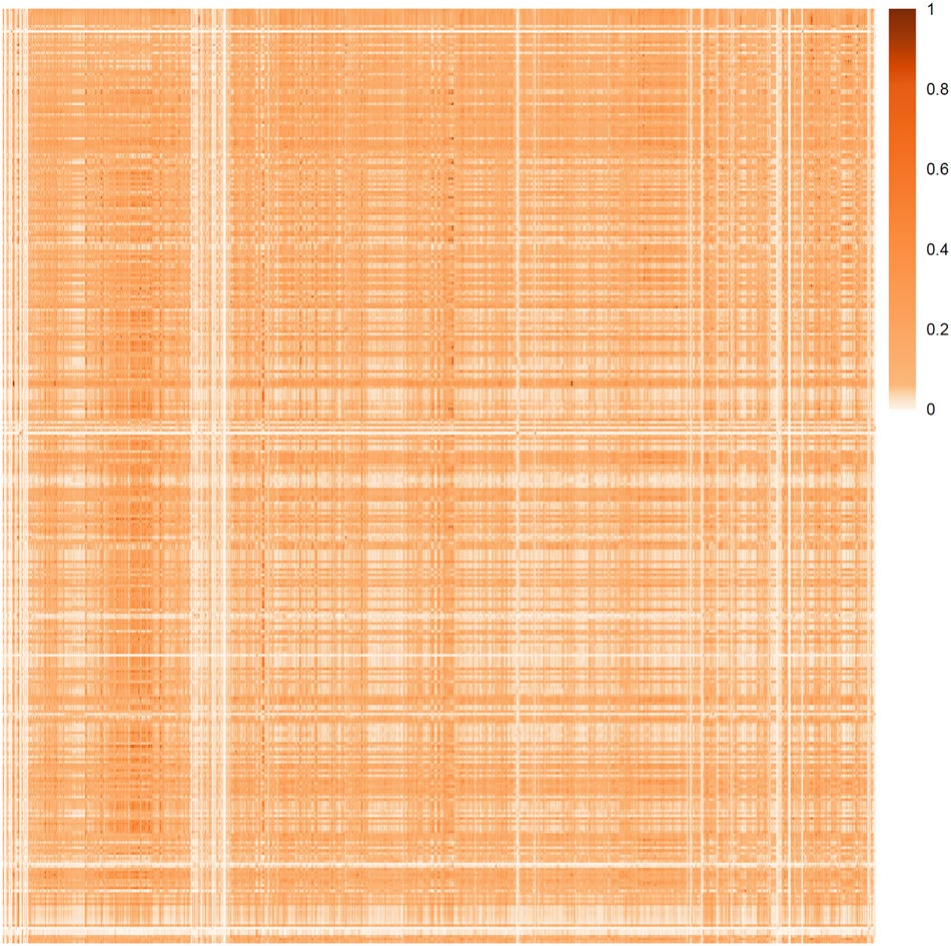
Heat-map corresponding to the Tanimoto coefficient based similarity of the DPCs with the drugs of the DrugBank: The heat-map corresponds to the molecular similarity of 349 DPCs and 2,159 drugs related to “drugbank_approved_structure” dataset, based on the Tanimoto score. The columns of the matrix represent DPCs and rows represent drugs.

“Similar compounds tend to generate similar biological effect” is the key concept that forms the foundation of medicinal chemistry^85^. Applying this idea in our study, we attempted to screen potential phytochemicals that may produce similar effects to the already existing drugs. Application of the similarity concept constitutes the third step of the protocol. For this, we first calculated the pairwise Tanimoto coefficient (TC) values of 349 DPCs against all the 2,159 drugs related to “drugbank_approved_structure” dataset of DrugBank. TC similarity values are presented in form of heatmap (Fig. 6). A subset of TC values of 241 DPCs (D1 dataset) with the approved drugs present in DrugBank (1,684 drugs, D dataset), is selected for further analysis. To identify novel molecules from the DPCs that may have the potential to exhibit similar effects as of the existing drugs, selection criteria comprising two similarity conditions was applied to all the pairs. As described in the Methods Section, a DPC was considered for further analysis, only if (i) it has TC value > 0.85 with any of the drug of D dataset^86^, (ii) it should not have the TC value = 1 and also should not have SMILES identical with any of the drugs in the D dataset.

**Figure 7:**
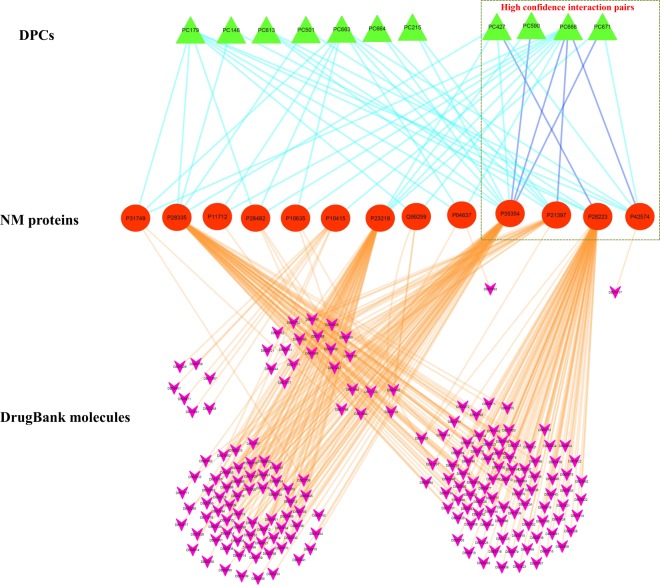
Tripartite network of NM proteins with their interacting DPCs and DrugBank molecules: The network consists of 212 nodes and 347 edges, consisting of interactions of 13 NM proteins (represented in the middle layer; red coloured nodes) with the 11 DPCs (represented in the top layer; green coloured nodes) and 188 drugs of the DrugBank (represented in the bottom layer; pink coloured nodes). Edges of the network are coloured differentially just for making distinctions among the interactions of DPCs and drugs with NM proteins. Edges representing interactions among DPCs and NM proteins are either represented in cyan (predicted by any 1 protein target algorithms) or blue (predicted by any 2 protein target algorithms). Six high confidence interaction pairs were identified in the network, consisting of the interactions of 4 DPCs with 4 NM proteins are represented in a rectangular box. No interaction pair predicted by all the methods appeared in the network. Interactions of the NM proteins with their associated drugs are represented with orange coloured edges.

Satisfying this selection criterion based on the above-discussed protocol, 11 DPCs of D1 dataset (PC179, PC146, PC613, PC501, PC663, PC664, PC215, PC427, PC590, PC668 and PC671) were successfully screened-in against 188 DrugBank compounds of D2 dataset (consisting of 467 drugs). These two sets of compounds were found to be connected via 13 NM proteins (P31749, P28335, P11712, P28482, P10635, P10415, P23219, Q99259, P04637, P35354, P21397, P28223, P42574). A tripartite network highlighting the interactions of 13 NM proteins with their corresponding 11 DPCs (from D1-dataset) and 188 drugs (from D2-dataset) shows that the multi-targeting effect of PC668 is noteworthy. As seen in Fig. 7, PC668 holds the potential to regulate 11 NM proteins of the network, which include three high confidence interactions with the P35354, P21397 and P42574. In addition to this, PC427, PC590 and PC671 also constitute the interaction partner of high-confidence pairs. Since these 11 DPCs maintain the drugability and similarity criteria used in this study, we propose these 11 DPCs for the detailed investigations by exploring their efficacies towards regulation of target protein activities *via in-vitro* and *in-vivo* experiments with a special focus on PC668, PC427, PC590 and PC67. The concept of designing synthetic analogues could also be explored to obtain satisfactory therapeutic effects from these DPCs.

#### Case study-II: Searching DPCs having poly-pharmacological similarity with AEDs

For the screening of DPCs that may have a tendency to elicit similar effects compared to the AEDs, a comparative analysis of the protein targets of both DPCs and AEDs was carried out. 40 AEDs were found to interact with 1,045 human proteins, forming 1,747 drug-protein pairs (listed in Supplementary File 12, Table S12). To recall, 349 DPCs were found to interact with 4,982 proteins. In the further analysis, we considered compound-protein interactions predicted by at least two of the three target prediction algorithms only. 607 interactions from DPCs and 161 from AEDs qualify this criterion.

**Figure 8:**
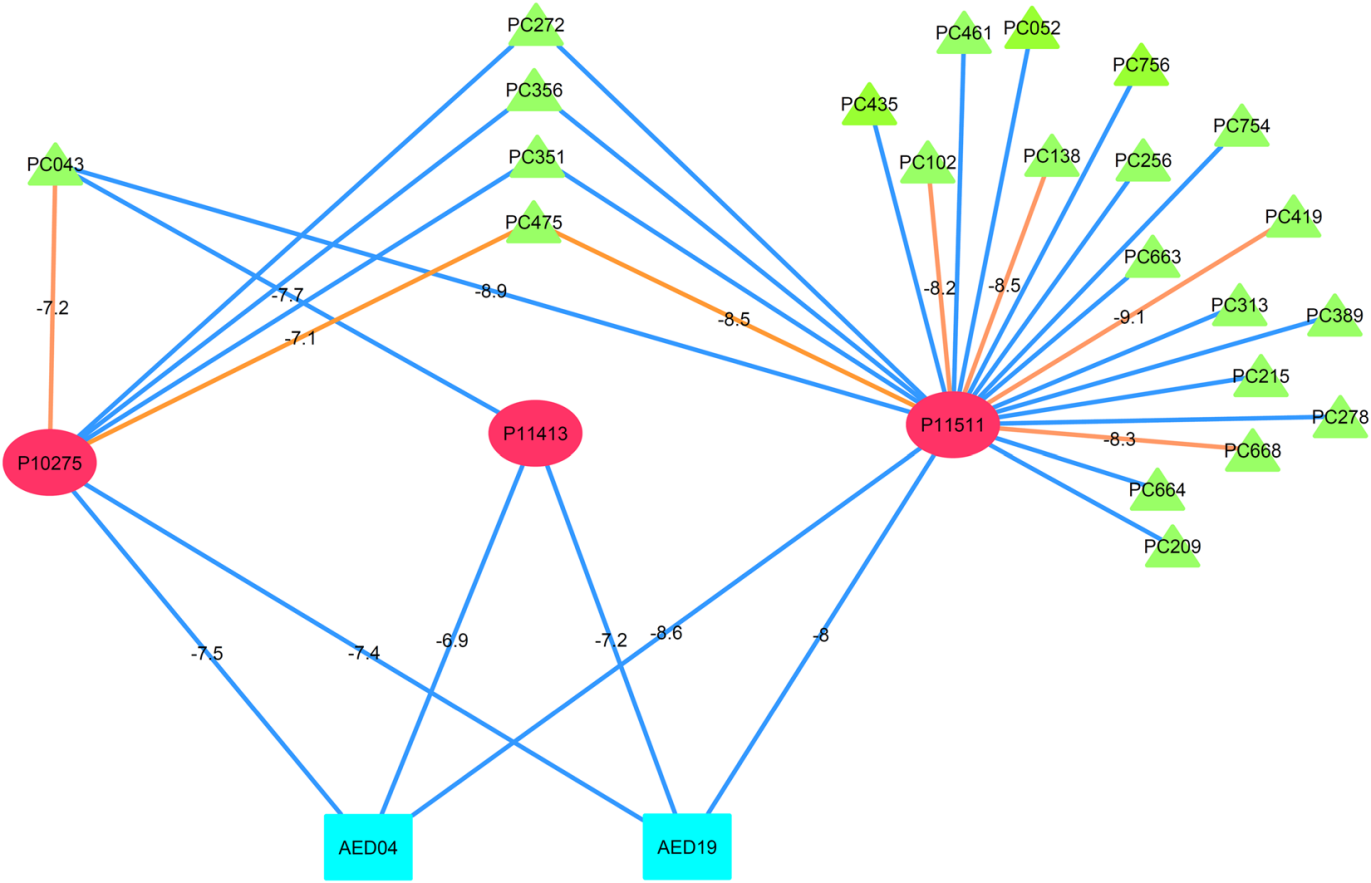
Multi-targeting potential of DPCs to regulate protein targets of AEDs: The tripartite network represents the multi-targeting potential of DPCs to regulate the protein targets of AEDs. Three protein targets (P10275, P11413 and P11511) commonly targeted by 22 DPCs (green triangular nodes) and 2 AEDs (cyan rectangular nodes) are represented in the middle layer of this tripartite network. The interactions of DPCs and AEDs with their protein targets are either represented as blue (predicted by any 2 protein target algorithms) or orange coloured edge (predicted by all 3 protein target algorithms). The binding energy values (kcal/mol) of the protein targets and their regulatory DPCs and AEDS are represented along with their corresponding edge in the network. Binding energy values show that these DPCs possess very good binding affinity for the protein targets of AEDs, in the range of −7.1 to −9.1 kcal/mol. PC043 is shown to target all the 3 proteins with binding energy values comparable to their corresponding AEDs, even better for few cases like AED19 and PC043 for P11413. For docking studies the following PDB IDs of proteins were used; 1XOW (P10275), 1QKI (P11413) and 3EQM (P11511). Here, binding energy calculations only for the high confidence DPC-PT interactions (*i.e*. predicted by 3 protein target algorithms) were considered.

35 proteins were common in both the datasets and were analysed further by constructing a tripartite network. This network consists of 260 interactions with 74 DPCs and 97 interactions with 20 AEDs, having 357 interactions in total (Supplementary Fig. 3 and Supplementary File 14, Table S14). AED19 and AED04 both are found to have interactions with three protein targets, namely P11511, P10275 and P11413. Association of these proteins is well studied in either direct correlation with epilepsy or with the onset of the seizure during epilepsy. When looked into the network, it is observed that the PC043 is a DPC that can regulate all the three protein targets of AED19 and AED04 (Fig. 8). Also to note, all of these three interactions of PC043 are returned by at least two of three target prediction algorithms.

P11511, an aromatase protein is a key enzyme of estrogen biosynthesis pathway. The role of aromatase inhibitors as a useful adjunct in the seizure control shows that the screening of regulatory DPCs for P11531 could be of significant importance in treatment procedures of EP, especially in male candidates^87,88^. Androgen receptor (AR), P10275 is highly abundant in several regions of the brain and the consequence of their activation by various androgens, like testosterone and its metabolites are responsible for the regulation of behavior and other neuronal functions. As reported earlier, changes in the expression of ARs by AEDs in the case of patients with temporal lobe EP shows that the modulation of ARs holds great potential in disease management^73^. The role of P11413 (Glucose-6-phosphate 1-dehydrogenase- an important enzyme responsible for glucose metabolism) in EP is based on the correlation of energy depletion and seizure development. Impairment in glucose metabolism has been examined in various epileptic patients and other epilepsy-related models^89^.

We would like to remark here that the network given in Supplementary Fig. 3 contains complete information about all the interactions among AEDs and DPCs with 35 protein targets, and therefore, any particular protein or group of proteins based on the aspects being explored may be chosen and their sub-network may be examined in detail. For example, in Supplementary Fig. 3, we have given two different sub-networks specific to only 2 protein-targets that may be analyzed further for docking and other detailed studies.

#### Case study III: Exploring the potential of DPCs to regulate a functional module in the EP-PPI network

**Figure 9:**
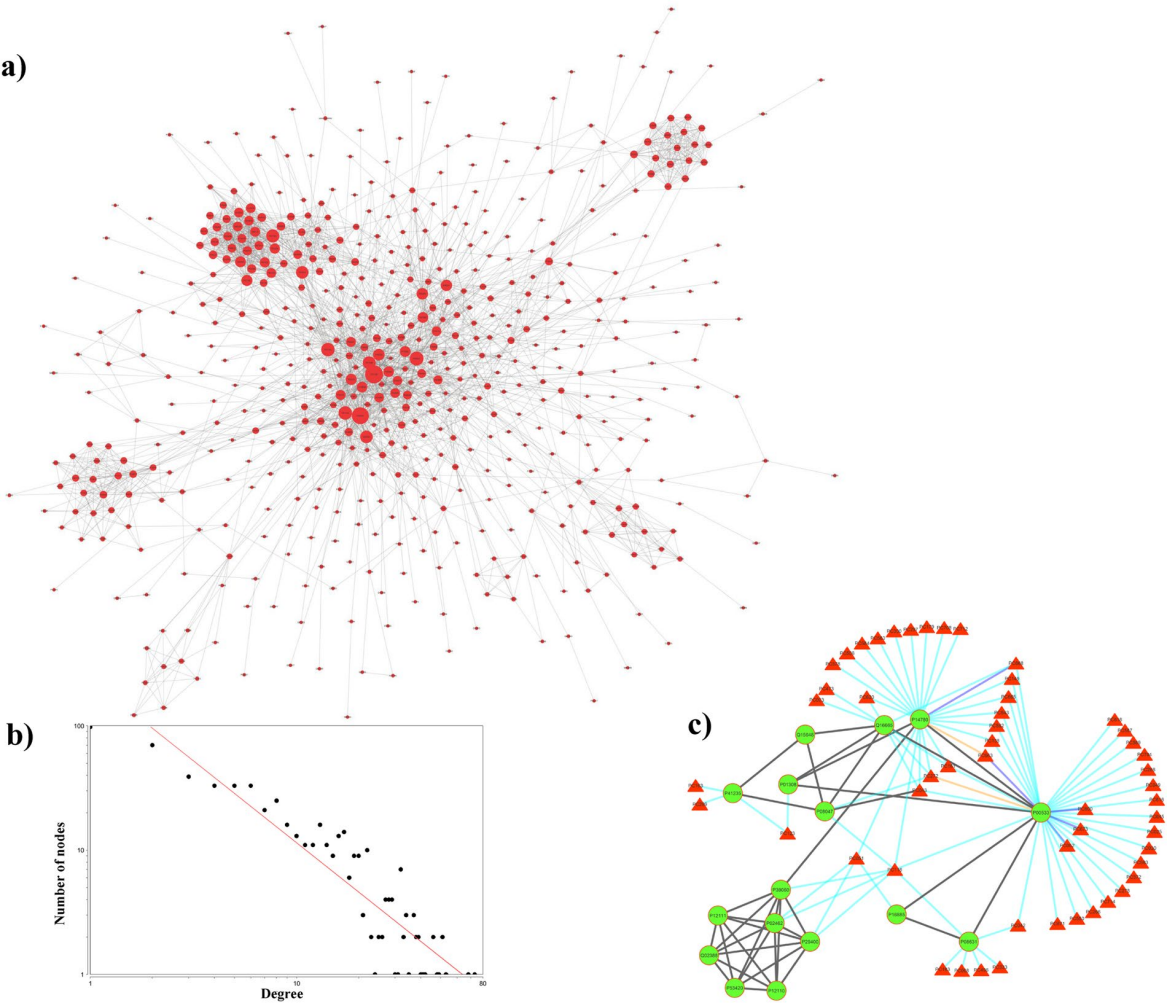
(**a**) EP-PPI network: The network (556 EP-pool proteins with 2,639 interactions among themselves) represents the sub-network of human PPI, obtained at the STRING confidence score of ≥0.9. The size of nodes is based on their corresponding degree value in the network. **(b**) Node degree distribution of EP-PPI network: Nodes connectivity in EP-PPI network is shown by plotting node-degree distribution on a log-log scale. Network follows a power-law degree distribution with *y* = 238.92*x*^−1.31^. **(c**) Interactions among the proteins of module 6 and their regulatory phytochemicals: 16 proteins of module 6 are represented as green nodes while the interactions among them are represented by black coloured edges. P14780 corresponding to gene MMP9 is directly known to regulate 3 other proteins of this module. Regulatory phytochemicals of proteins are represented as red coloured triangular nodes and added to the module from PC-PT network. Interactions among phytochemical-protein pairs are either represented as cyan (prediction from any 1 target prediction algorithms), blue (prediction from any 2 target prediction algorithms) or orange coloured edges (prediction from all 3 target prediction algorithms).

To investigate the comprehensive effects of EP proteins on the entire human PPI, a sub-PPI regulated by EP proteins was constructed and analysed. For that, a high confidence human PPI network was constructed using a STRING score of ≥0.9 in which a total of 794 EP proteins could be mapped (Supplementary File 13, Table S13). Out of these, 611 EP proteins were showing 2,681 interactions with other EP proteins (*i.e*. these 611 own the tendency to regulate each other) while remaining existed as individual nodes. Isolated nodes were removed and the giant component of the network consisting of 566 nodes and 2,639 edges (referred to as EP-PPI network) was considered for the further analysis (Fig. 9a). The network degree distribution shows a power-law with *y* = 238.92*x*^−1.31^ (Fig. 9b).

To get an insight about the overall organization and the inter-relationship of EP proteins in the EP-PPI network, this network was subjected for modularity analysis using MCODE algorithm that returned 21 densely connected regions *i.e*. clusters. To assess the biological role of each identified cluster, gene ontology (GO) based enrichment of the genes associated with various clusters were examined using DAVID. The complete description of the identified clusters, proteins associated with them and their GO-based annotation is given in Supplementary File 13, Table S13.

As shown in Table S9, the clusters are found to be involved in multiple biological processes, majority being associated with metabolism and signalling events. Proteins of modules 3, 6, 7 and 9 were directly or indirectly linked with the metabolic processes. For example, module-6 includes one of the major key players of the epileptogenesis, *i.e*. MMP9 (P14780) gene that has a special role in temporal lobe epilepsy^90^ and also in the cases of intractable epilepsy^91^. In order to gain the information of regulatory potential of MMP9 with respect to other EP proteins, position of this node was checked in both EP-PPI network and its corresponding module. In EP-PPI, its C_d_ value is 20 implying that MMP9 can regulate 19 other EP proteins of this network, while in its respective module (module-6) it shows direct links with 3 of these 19 proteins. This protein was also assessed for an important network measure known as betweenness centrality (C_b_), which reflects the frequency of a node to lie on the shortest path between all the node pairs of a network^92^. Network analysis shows that P14780 holds the maximum C_b_ value of 0.53 among all the other proteins of module-6, thereby reflecting the key relevance of this protein in terms of its information spreading capacity. Discerning the regulatory importance of MMP9 in this module, we found it essential to identify the DPCs that have potential to regulate the activities of MMP9 because the protein lies in the center of this module and regulation at this level will tend to generate global effects on the entire module. For detailed investigation, a sub-network corresponding to module-6 and its regulatory DPCs was constructed (Fig. 9c). As shown, 22 DPCs are having interactions with the proteins of this module, amongst those 13 DPCs are multi-targeting that interact with MMP9 and other proteins also. DPCs corresponding to PC663 (luteolin) and PC668 (apigenin) are found to possess multi-targeting capacity at a high confidence score, thereby indicating the scope for their future investigation. In addition, roles of PC272 and PC043 may also be explored in detail as both of them are found to directly regulate 4 proteins of the module. Using the similar approach other key proteins and their regulators at the module level could be identified and studied in detail.

In the following, we briefly discuss the proteins involved in other modules that are enriched in metabolic processes (that are 3, 7 and 9). Proteins of module-7 are generally associated with folic acid metabolism. Folic acid is known to have significant importance in the health of epileptic patients, due to various vascular and neurological conditions that appear in its deficient state^93^. Module-3 is rich in genes associated with the cytochrome P450 family like CYP2C9, CYP3A4, CYP2E1 etc. Increased expression of these genes (like CYP2E1) has been observed in the conditions of spontaneous recurrent seizures^94^. Also, pharmacogenetic effects of cytochrome P450 on various disease states, especially CYP2C9 in epilepsy have been established in previous studies^95^. The role of proteins of module 9 in epilepsy is established based on various studies according to which regulation of glutamate homeostasis is of key importance in the disease process. Glutamate is a prominent excitatory amino acid and impaired transport system of the same has been observed in the condition of epilepsy^96^. Further, in maintaining glutamate homeostasis, glutamate-astrocyte system is reported to play an important role in disease pathogenesis^76^.

Proteins of module-1 and module-8 are majorly involved in G-protein coupled receptor signaling pathway. These clusters were highly enriched in genes like GRM (glutamate metabotropic receptors), NPY (neuropeptide Y receptors) and HTR (hydroxytryptamine receptors). NPY receptors are densely located in the region of strata radiatum of brain and their altered expression in epileptic brain tissues have been noted earlier^97^. Role of HTR receptors in epilepsy is mainly through their participation in the serotonergic neurotransmission process. Other signaling events associated proteins are from module 4, 5 and 11 corresponding to GO related to gamma-butyric acid signaling, MAPK cascade and signal transduction respectively. Gamma butyric acid (GABA) is one of the important and most widely studied classes of neurotransmitters in relation to epilepsy. GABA receptors are the well-known markers for the disease and the role of GABAergic transmission in epileptogenesis is well studied^98^. Since a variety of gene regulatory events are involved in the onset of this disease, a single event is not considered responsible for disease development. Recently, the roles of MAPK targets have been successfully proved in regulating the synaptic excitability that appears in epileptic conditions^99^.

Since the underlying mechanisms for governing the onset of epilepsy are diverse in nature, a similar trend has been observed at the modular analysis of the EP-PPI. Though the proteins associated with epilepsy are involved in various biological processes based on GO terms, majority of them are focused on the metabolic and signaling events of the cell.

The complete information of the data used and the results generated in this study is summarised in Table 2.

**Table 2:** Summary of the data used and results generated in this study.

Serial No.	Particulars of the data used and the results generated	Associated numbers	Reference
1.	Anti-epileptic herbs (AEHs)	63	Table 1
2.	Anti-epileptic drugs(AEDs)	40	Supplementary File 4, Table S4
3.	Unique phytochemicals (PCs) identified in considered AEHs	867	Supplementary File 5, Table S5
4.	PCs and their AEHs association	63 AEHs and 867 PCs association	Supplementary File 6, Table S6
5.	Drugable phytochemicals (DPCs) and their ADMET properties	349	Supplementary File 2, Table S2
6.	Chemical classification of DPCs	16 broad chemical classes	Supplementary File 7, Table S7
7.	Human protein targets of DPCs	4,982	Supplementary File 9, Table S9
8.	Epilepsy targets(EP-gene pool)	1,179	Supplementary File 1, Table S1
9.	DrugBank approved drugs used for Tanimoto coefficient(TC) calculations with DPCs	2,337	Supplementary File 3, Table S3
10.	DrugBank approved protein targets	2,669	Supplementary File 3, Table S3
11.	High confidence DPC–PT pair	66 (predicted by all 3 target prediction algorithms)	Supplementary File 8, Table S8
12.	DPC–PTE pairs (Drugable phytochemicals with their epilepsy protein targets)	3,002	Supplementary File 8, Table S8
13.	Human pathways associated with 4,982 protein targets of DPCs	314	Supplementary File 9, Table S9
14.	Human protein targets of DPCs (out of 4,982) having KEGG pathway association	3,030	Supplementary File 9, Table S9
15.	PT–HP pairs (Human protein targets of DPCs and their associated human pathways)	15,535	Supplementary File 9, Table S9
16.	PTE–HP pairs (Human protein targets of DPCs specific to epilepsy and their associated human pathways)	2,862	Supplementary File 10, Table S10
17.	KEGG pathways associated with nervous system	10	Supplementary File 11, Table S11
18.	Human targets of DPCs associated with 10 nervous system pathways; referred as “NM proteins”	100	Supplementary File 11, Table S11
19.	Mapped NM proteins onto the DrugBank approved protein target list	81	Supplementary File 11, Table S11
20.	Human protein targets (from DPC-PT network) associated with 81 NM proteins; D1 dataset	241	Supplementary File 11, Table S11
21.	Drugs (from DrugBank database) associated with 81 NM proteins; D2 dataset	467	Supplementary File 11, Table S11
22.	Approved drugs of DrugBank constituting D dataset	1,684	Supplementary File 11, Table S11
23.	NeuMoDs	11	Supplementary File 11, Table S11
24.	mGluRs mapped in the DPCs protein targets list	8	Supplementary File 11, Table S11
25.	Regulatory DPCs of mGluRs	11	Supplementary File 11, Table S11
26.	Human protein targets of 40 AEDs	1,045	Supplementary File 12, Table S12
27.	AEDs and their human protein target pairs	1,747	Supplementary File 12, Table S12
28.	High confidence AEDs and their human protein target pairs	161	Supplementary File 12, Table S12
29.	Epilepsy proteins mapped in human PPI network of STRING score ≥900	794	Supplementary File 13, Table S13
30.	Proteins in EP-PPI network	556	Supplementary File 13, Table S13
31.	Protein targets – AED/DPC pair (Case study II)	357	Supplementary File 14, Table S14

## Summary and Future Prospects

Despite the recent developments in preclinical research that have facilitated the scientific community to discover valuable and effective drugs against epilepsy, occurrence of the seizures is neither accurately predictable nor under control in more than one-third of the patients affected by epilepsy. Traditional Indian medicinal system has always remained a great source of information about the implications of various herbs and herbal formulae for the management of various diseases. Though the usage of herbal medicines in epileptic treatment is well known for several thousand years, lack of robust confirmation regarding their efficacy has limited their usage in the present scenario. Therefore in this study, we have examined the anti-epileptic potential of various traditional Indian herbs using network pharmacology approach towards discovery of novel drug-like molecules. An extensive search about the anti-epileptic herbs prescribed in Ayurveda resulted in the identification of 63 herbs; detailed information about the phytochemicals content in these herbs forms the basis of present study. To reveal component level anti-epileptic effects of the selected herbs, these were subjected to database screening for identification of their phytochemical composition. Out of total 1,993 phytochemicals collected, 867 unique entries were identified based on their chemical information. 349 phytochemicals from this list of 867 are found to have the drug-like properties and are reported as putative drug-like phytochemicals (DPCs). The polypharmacological effects of DPCs are evaluated on the basis of their human-protein targeting capability. To limit the phytochemical-protein interactions specific to epilepsy, an epilepsy gene pool comprising of 1,179 genes (EP-pool) is constructed. Only 336 DPCs show the potential to interact with the proteins of EP-pool. P10636 (*i.e*. microtubule-associated protein tau) is highlighted as one of the majorly targeted proteins by the DPCs (95 in number) of AEHs. Eleven potential regulators of mGluRs with their supporting docking and interaction analyses are highlighted in this work. Among these, 3 DPCs *i.e*. PC146 (caproic acid), PC501 (valeric acid) and PC179 (butyric acid) also show similarity with DrugBank molecules, thereby indicating their future usage in mGluR5 and mGluR1 regulators design strategies. Role of EP33 (*Glycyrrhiza glabra*) and EP47 (*Punica granatum*) as the major sources of potential drug-like phytochemicals having mGluRs regulatory potential in epilepsy is explained. Four DPCs namely, PC663 (luteolin), PC664 (pinocembrin), PC251 (pectin) and PC668 (apigenin) that successfully passed through the DrugBank molecules similarity criterion have shown the propensity to interact with all the protein targets of two AEDs. Among the above mentioned 4 DPCs, PC663 and PC668 are found to regulate the module-6 of EP-PPI network either by directly interacting with the protein P14780 (having highest C_b_ value) or its neighbors. The presence of high confidence interactions of PC668 (apigenin) in both the case studies corresponding to DrugBank molecules and AEDs, establishes a strong regulatory potential of this DPC against epilepsy. The presence of this DPC in both EP33 and EP47 further strengthen the anti-epileptic action of these herbs. The multi-therapeutic effects elicited by PC043 (17-beta estradiol) are predicted as it is found to simultaneous interact with all the 3 proteins targets of AEDs. It is interesting to note that some DPCs, when analysed for their binding energy values, show much lower binding energy for the protein-targets compared to their corresponding AEDs.

Comparison of the screened DPCs having multi-targeting and/or synergistic effects with the existing drugs of DrugBank and currently used anti-epileptic drugs performed in this study will be a substantially important resource for future anti-epileptic drug design strategies. Also, the representation and analysis of complex DPC-PTE interactions and DPC-Pathway associations in the form of network maps will be helpful to the researchers working in this area to further explore this deluge of information in a relatively simple mode. Towards explicating the complete regulatory map of phytochemical space in epilepsy management, one aspect that is not covered in this work is the regulation of functional ncRNAs. Clinical utility and pathophysiology of ncRNAs (noncoding RNAs) in epilepsy are being continuously reported in recent years with various studies supporting the role of these regulatory molecules in disease pathogenesis and therapeutics. Different classes of ncRNAs including small sized ones like miRNAs as well as longer ones like circRNAs have been reported to have differential expression patterns in the epileptic patients^100^. In recent years, several approaches have been developed to investigate the associations between small compounds and miRNAs^101–103^. We believe that the application of similar approaches to explore the phytochemicals’ anti-epileptic potential *via* regulating miRNAs and cirRNAs will pave the way towards the deeper understanding of disease pathogenesis and its associated therapeutic studies. Although, the aspects of ncRNAs as potential targets have not been explored in this study, we believe that integrating the concepts of network pharmacology developed in this work to examine the natural compounds for their ncRNAs (like miRNAs, circRNAs etc.) regulatory prospects will be helpful.

One of the main outcome of the present study is the identification of 11 NeuMoDs (PC179, PC146, PC613, PC501, PC663, PC664, PC215, PC427, PC590, PC668 and PC671) effective in regulating 13 neuromodulatory proteins. Moreover, regulation of mGluR5 by the caproic acid and valeric acid has also been highlighted in this work. We were also able to report 74 DPCs having potentially similar polypharmacological activities to that of drugs which are either approved or under clinical trials against epilepsy. Although the present work is completely based on computational studies, the developed methodology is quite comprehensive and adoption of stringent selection criteria have allowed the identification of high confidence phytochemical – protein-target pairs with their multi-targeting and synergistic actions on epileptic genes. Augmenting these computational results with the experimental studies in future will be of great help towards developing a better and deeper context of the underlying mechanisms of Epilepsy. We are hopeful that the developed protocol will help in the development of a broad understanding of complex biological associations particularly in the cases of neurological diseases and disorders. Also, the hierarchy of steps used in this computational framework will be immensely helpful to delineate the phytochemical-level anti-epileptic potential of the herbs of not only Indian Ayurvedic system but other traditional medicinal systems of the world also in a much clearer, detailed and meaningful manner against any class of disease or disorder. This study may be considered as a major step towards integrating the wealth of traditional knowledge with scientific outlook for their application in modern day therapeutics to meet the current demand of drug-discovery.

## Supplementary information

Supplementary Dataset 1

Table S1

Table S2

Table S3

Table S4

Table S5

Table S6

Table S7

Table S8

Table S9

Table S10

Table S11

Table S12

Table S13

Table S14

## Data Availability

All the datasets generated or analysed in this study are provided in the Supplementary Files. The preprint version of this manuscript along with entire data is available at bioRxiv with doi: https://doi.org/10.1101/471474.
