# Supplementary material for: Insights about multi-targeting and synergistic neuromodulators in Ayurvedic herbs against epilepsy: integrated computational studies on drug-target and protein-protein interaction networks
Source: Sci Rep. 2019 Jul 22;9:10565. doi: 10.1038/s41598-019-46715-6 (PMC6646331; doi:10.1038/s41598-019-46715-6)
Supplement: Supplementary file 1 — Supplementary Dataset 1 [file 41598_2019_46715_MOESM1_ESM.pdf]

Supplementary Figures

Insights about multi-targeting and synergistic neuromodulators in Ayurvedic herbs against epilepsy: integrated computational studies on drug-target and protein-protein interaction networks

Neha Choudhary, Vikram Singh*

Centre for Computational Biology and Bioinformatics, School of Life Sciences, Central University of Himachal Pradesh, Dharamshala, India -- 176206.

*Corresponding author: vikramsingh@cuhimachal.ac.in

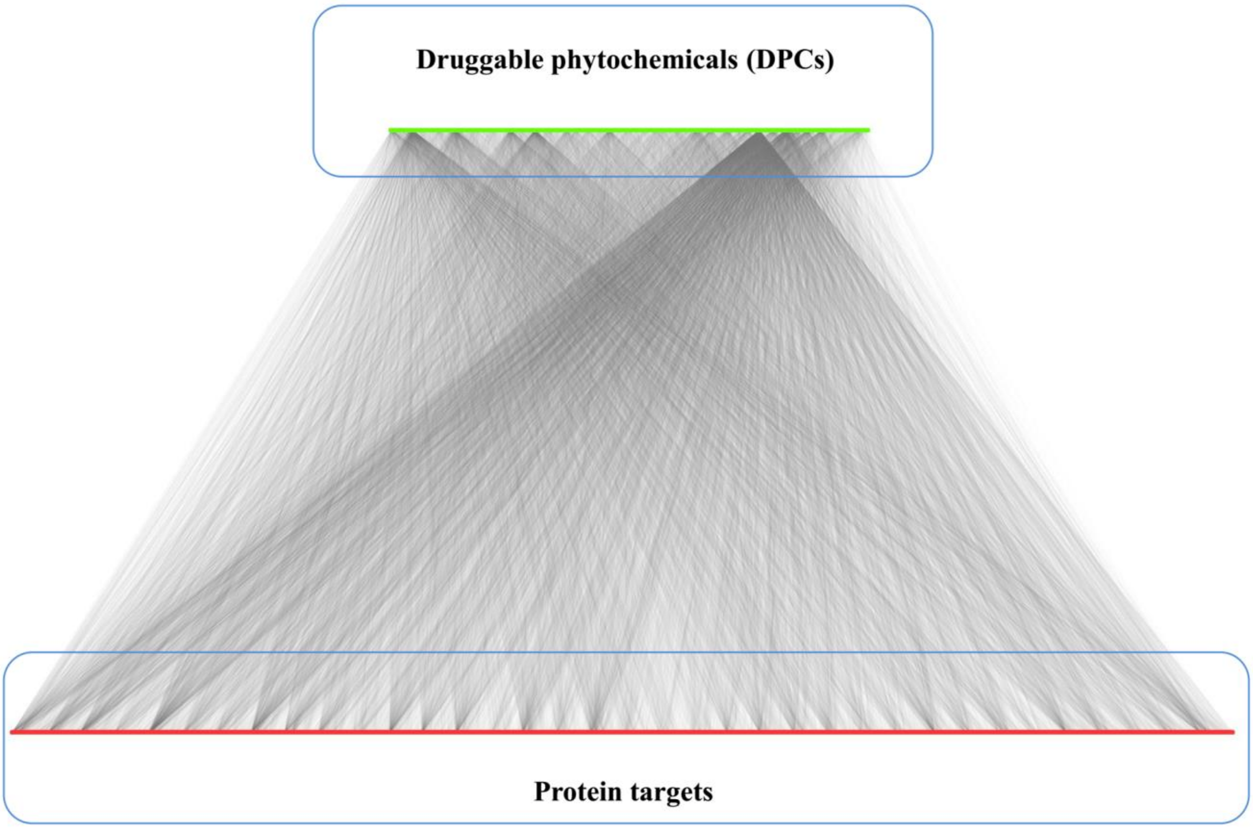

Fig. S1 Bipartite DPC-PT network consisting of 349DPCs and their 4982 protein targets.

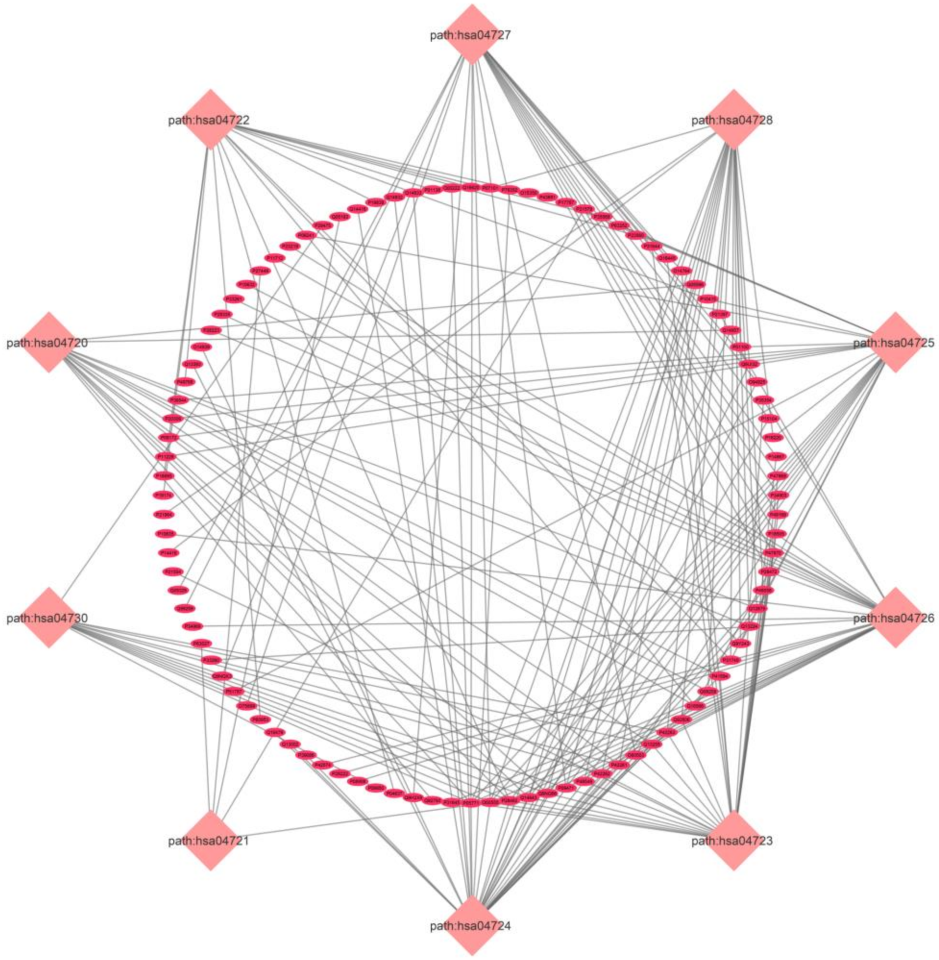

Fig. S2 This subnetwork consists of 110 nodes and 200 edges corresponding to 10 KEGG pathways associated with nervous system namely, glutamatergic synapse (path:hsa04724), GABAergic synapse (path:hsa04727), cholinergic synapse (path:hsa4725), dopaminergic synapse (path:hsa4728), serotonergic synapse (path:hsa4726), long-term potentiation (path:hsa04720), long-term depression (path:hsa4730), retrograde endocannabinoid signaling (path:hsa04723), synaptic vesicle cycle (path:hsa04721) and neurotrophin signaling pathway (path:hsa4722) and their associated 100 proteins. Pathways are represented in diamond-shaped nodes while proteins in circular nodes.

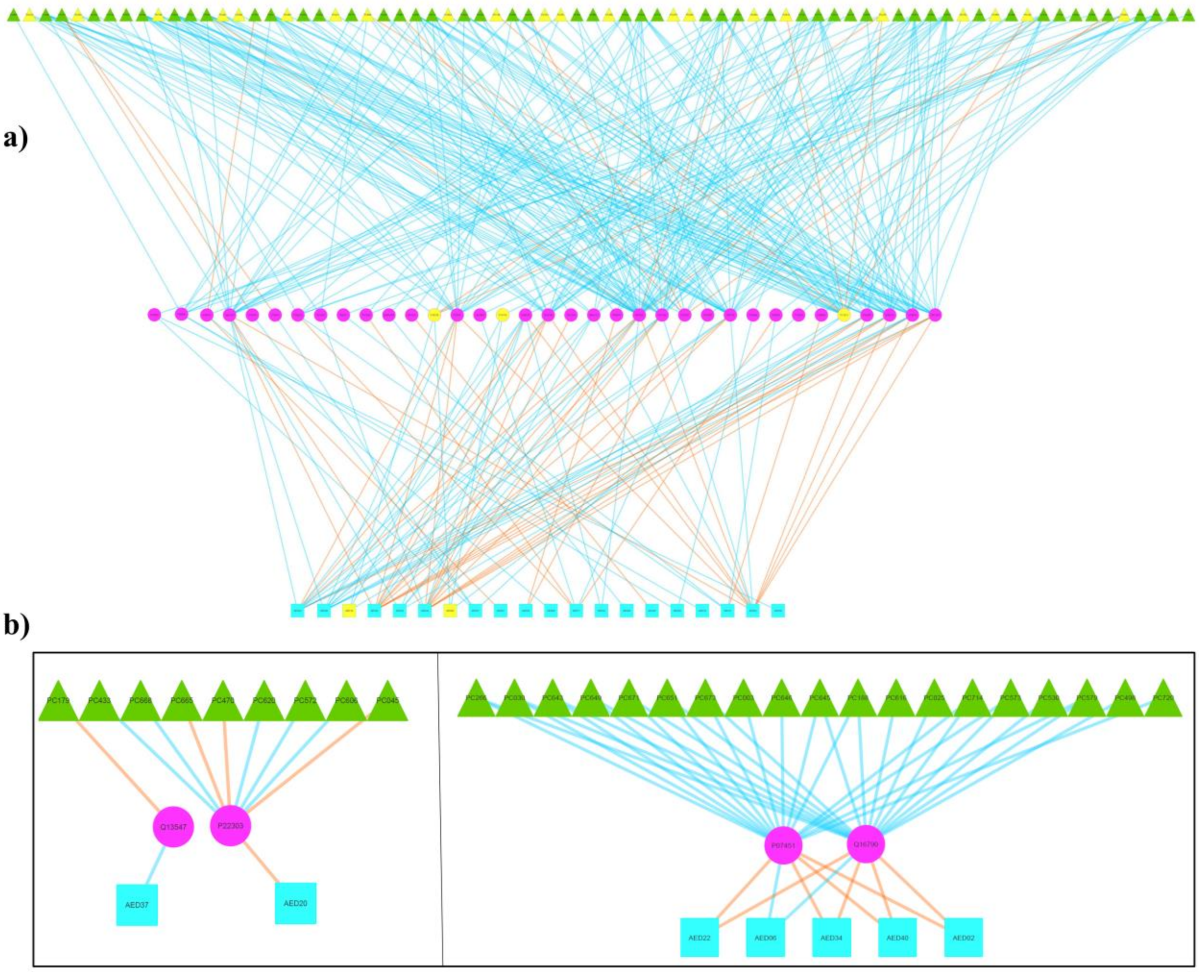

Fig. S3 a) The sub-network specific to the 35 common proteins and their regulatory DPCs and AEDs. The tripartite network consists of 357 interactions where 260 interactions came from 74 DPCs and 97 interactions with 20 AEDs. **b)** Sample sub-networks derived from the above network.

Yellow highlighted nodes in the network represent the sub-network considered for the case study as shown in Figure 8 of the manuscript.
